# Endogenous retrovirus-K promoter: a landing strip for inflammatory transcription factors?

**DOI:** 10.1186/1742-4690-10-16

**Published:** 2013-02-09

**Authors:** Mamneet Manghera, Renée N Douville

**Affiliations:** 1 Department of Biology, The University of Winnipeg, Winnipeg, MB, Canada; 2 Department of Immunology, University of Manitoba, Winnipeg, MB, Canada

**Keywords:** Endogenous retrovirus (ERV), Long terminal repeat (LTR), Transcription factor, Inflammation, Promoter, Interferon-stimulated response element (ISRE), Nuclear factor κB (NF-κB), Human Immunodeficiency Virus (HIV).

## Abstract

Humans are symbiotic organisms; our genome is populated with a substantial number of endogenous retroviruses (ERVs), some remarkably intact, while others are remnants of their former selves. Current research indicates that not all ERVs remain silent passengers within our genomes; re-activation of ERVs is often associated with inflammatory diseases. ERVK is the most recently endogenized and transcriptionally active ERV in humans, and as such may potentially contribute to the pathology of inflammatory disease. Here, we showcase the transcriptional regulation of ERVK. Expression of ERVs is regulated in part by epigenetic mechanisms, but also depends on transcriptional regulatory elements present within retroviral long terminal repeats (LTRs). These LTRs are responsive to both viral and cellular transcription factors; and we are just beginning to appreciate the full complexity of transcription factor interaction with the viral promoter. In this review, an exploration into the inflammatory transcription factor sites within the ERVK LTR will highlight the possible mechanisms by which ERVK is induced in inflammatory diseases.

## Review

### Background

The human genome contains thousands of genetic parasites called endogenous retroviruses (ERVs) (reviewed in [1]). These genomic invaders endogenated through infection of germ-line cells; this gave rise to gametes containing integrated proviruses and viable progeny in a symbiotic relationship with the virus. The symbiogenesis between the human genome and these DNA parasites has been a major contributing factor to genetic and transcriptional changes during hominid evolution [2,3]. Some ERVs confer biological benefits to humans, and have been retained in our genome for a considerable period of time. For instance, the *env* (envelope) genes of ERVW encode syncytin proteins which contribute to the differentiation of syncytiotrophoblast in chorionic villi, aiding in normal placental development during pregnancy [4,5]. At the same time other ERVs, notably ERVK, may be deleterious to the host considering its capacity to express viral RNA, proteins, and under select conditions, intact virions. Isolation of mature ERVK virions from primary cancer cells and cell lines reveals expected genomic viral RNA and proteins, although infectivity has yet to be demonstrated empirically [6,7]. Thus, the pathological role of ERVK remains speculative [8-10]. It is clear however, that ERVK is transcriptionally active in inflammatory diseases including Rheumatoid Arthritis (RA) [11], Systemic Lupus Erythematosus (SLE) [12], Schizophrenia [13], Amyotrophic Lateral Sclerosis (ALS)[14], and multiple types of cancers [15]. Several infectious diseases are also characterized by enhanced ERVK expression, including Human Immunodeficiency Virus (HIV) infection [16,17]. These associations are suggestive of shared mechanisms by which ERVK expression may be regulated under inflammatory conditions. Epigenetic factors can play a large role in the control of these retroelements, and are reviewed extensively elsewhere [18]. In contrast, this review aims to highlight the current literature in regards to cellular transcription factors which modulate ERVK expression by interacting with ERVK long terminal repeats (LTRs).

### Importance of the LTR in driving ERVK expression

**Figure 1:**
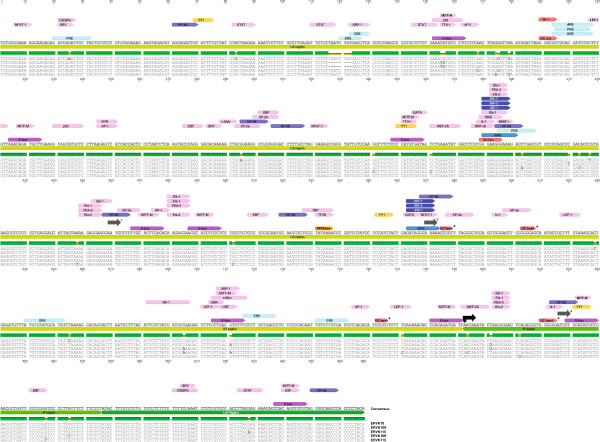
***In silico *examination of the conserved transcription factor binding sites and response elements within five endogenous retrovirus-K (ERVK) 5**^**′**^**-LTRs using ALGGEN-PROMO software **[25]**.** The ERVK LTR consensus sequence was constructed using individual ERVK LTRs in the following order (GenBank accession numbers in brackets): ERVK-10 (M12854.1), ERVK-9 (former HERV-K109) (AF164615.1), ERVK-8 (former HERV-K115) (AY037929.1), ERVK-6 (former HERV-K108) (AF074086.2) and ERVK-113 (JF742069.1). GC boxes 1 to 4, indicated by asterisks, were adapted from [22]. Abbreviations used include: PRE = Progesterone Response Element, CRE = CREB Response Element, ERE = Estrogen Response Element, ARE = Androgen Response Element, E-box = Enhancer Box, GRE = Glucocorticoid Response Element, ISRE = Interferon-Stimulated Response Element, GC box, GA box, ORE = Oct Response Element. Conventional (793bp) [22] and three alternative (460, 570, and 826 bp) [21,23] transcriptional start sites are depicted by black and grey arrows, respectively. Sequence alignment and annotation were performed using Geneious software [26].

The gene expression of ERVK is under the direct control of its long terminal repeats (LTRs). The 5^′^ LTR promotes sense transcription of the viral genome (Figure 1). It remains unclear if the 3^′^ LTR influences anti-sense transcription of the provirus, as seen with other retroviruses such as HIV and HTLV-1 [19-21]. Each flanking viral LTR consists of a U3, R, and U5 regions in 5^′^ to 3^′^ direction. U3 region is the most important as it contains all the sequences – TATA independent promoter, enhancers, and transcription factor binding sites – required for initiation of transcription of ERVK genes. Additionally, alternative transcriptional start sites have been proposed [21-23], perhaps allowing for differential transcripts under a variety of physiological conditions. ERVK LTR subgroups exhibiting specific base insertions may also influence the transcriptional regulation of these elements [24]. We are just beginning to appreciate the full complexity of ERVK transcriptional regulation, and despite a full understanding, it is clear that both cellular transcription factors and virally encoded proteins can be transcriptional activators of the ERVK LTR. A complete view of the putative transcription factor binding sites and regulatory elements within the ERVK LTR (Figure 1) allows us to confirm and speculate upon the involvement of several transcription factors in the pathogenesis of inflammatory diseases through induction of ERVK gene expression.

### Overcoming epigenetic silencing of ERVK

ERVs have been transcriptionally silenced over evolutionary time from accumulation of point mutations and deletions. Additionally, epigenetic mechanisms, particularly CpG methylation and cytosine deamination of the LTR, control the basal expression of ERVK in various cell types and tissues; these have been extensively reviewed elsewhere [18]. The methylation status of U3 region of the ERVK LTR has been shown to correlate with its transcriptional activity; low levels of LTR methylation have been shown to result in high levels of ERVK expression [27,28]. Methylation of CpG dinucleotides in genomic DNA serves to repress transcription of genes by interfering with the binding of sequence-specific transcription factors [27]. Similarly, CpG methylation of the ERVK LTR provides a natural defence against intragenomic parasites such as ERVK.

The activity of ERVK LTRs has been further repressed by deamination of methylated CpG dinucleotides. Spontaneous deamination is a major source of abundant G to A and C to T mutations observed in many ERVK LTRs [29], which render them incapable of transcription. GC box 1 and GC box 4 in the LTR (Figure 1) exhibit G to A and C to T conversions, respectively [29].

APOBEC3G-mediated cytidine deamination is another common source of G to A, and less frequently C to T, mutations in the ERVK LTR [29]. This process occurs after retroviral infection but prior to the integration of proviral DNA within the human genome [29]. In many ERVK LTRs, APOBEC3G has been responsible for excessive G to A mutations [29-31]. APOBEC3G targets GG dinucleotides as well as GGG trinucleotides, efficiently mutating Tryptophan codons [29]. G to A mutation of Tryptophan codons generates new stop codons, which is lethal to ERVK when present within coding regions. However, when this mutation is present within non-coding regions, it can prevent the binding of crucial transcription factors recognizing that region. In fact, GC box 2 (Figure 1) on the ERVK LTR displays a plus-strand GG to AG change [22,29,31], which likely inhibits the binding of Sp1 and Sp3 transcription factors to this GC box.

Despite the layers of epigenetic control, basal and inducible ERVK expression is evident in normal physiology and inflammatory disease [32,33], indicating that additional regulatory machinery is involved in ERVK transcription. Together, cell type-dependent epigenetic programming and expression of transcriptional regulatory factors are expected to guide the overall ERVK transcriptome, and is apt to become critical in disease states where methylation-mediated silencing is compromised.

### Influence of cellular transcription factors on ERVK expression

Other than epigenetic factors, cellular transcription factors are also crucial for regulating the activity of the ERVK LTR. Different types, expression levels and activity of key transcription factors may be required to achieve distinct tissue-specific ERVK expression. However, relatively little is known about the transcriptional regulation of ERVK when pertaining to tissue specificity, conditions of inflammation and disease states. Interestingly, many ERVK LTRs have intact and conserved binding sites for human transcription factors (Table 1), yet few transcription factors have been experimentally shown to modulate ERVK LTR activity (Table 2).

**Table 1:** Cellular transcription factors predicted to bind to their respective putative consensus sequences on the ERVK LTR

**Transcription Factor**	**Consensus sequence**	**References**
Activating Protein 1 (AP-1); c-Jun/c-Fos	TGA(G/C)TCA	[34]
Activating Protein 2 (AP-2α)	GCCNNNGGC	[35]
Androgen receptor (AR)	GG(A/T)ACANNNTGTTCT (ARE)	[36]
cAMP Response Element Binding (CREB) protein	T(G/T)ACGTCA (CRE)	[37]
cAMP Response Element Modulator (CREM-α)	T(G/T)ACGTCA (CRE)	[37]
CCAAT-Enhancer Binding Protein (C/EBP)	N(A/G)CCAAT	[38,39]
Cellular Myeloblastosis virus protein (c-Myb)	(T/C)AAC(G/T)G	[40]
Cellular Myelocytomatosis virus protein (c-Myc)	NNNCACGTGNN (E-box)	[41,42]
Early B-cell Factor (EBF)	CCCNNGGG	[43]
Estrogen Receptor (ER-α; ER-β)	GGTCANNNTGACC (ERE)	[44]
E-twenty six (ETS-1; ETS-2)	GGA(A/T)	[45]
ETS-like Transcription Factor 1 (Elk-1)	GGA(A/T)	[45]
GATA binding protein (GATA)	(A/T)GATA(A/G)	[46]
Glucocorticoid Receptor (GR-α; GR-β)	GGTACANNNTGTTC (GRE)	[47]
Ikarose-1 (Ik-1)	TGGGA(A/T)	[48]
Interferon Regulatory Factor (IRF-1; IRF-3; IRF-7)	GAAANN repeats (ISRE)	[49,50]
Lymphoid Enhancer-binding Factor 1 (LEF-1)	CTTTGAA	[51]
Microphthalmia-associated Transcription Factor**-**M (MITF-M)	CA(C/T)GTG (E-box)	[23]
Monocyte Enhancer Factor-2 (MEF-2A)	CT(A/T)(A/T)AAATAG	[52]
Myc Associated Zinc finger protein (MAZ)	GGGAGGG	[53]
Nuclear Factor of Activated T cells (NFAT-1)	GGAGAA	[54]
Nuclear Factor I (NF-I)	TTGGCNNNNNGCCAA	[55]
Nuclear Factor Kappa B (NF-kB)	GG(G/A)(G/A)NN(C/T)(C/T)CC	[56]
Octamer-1 (OCT-1)	ATGCAAAT (ORE)	[57]
Polyomavirus Enhancer Activator 3 (PEA-3)	GGA(A/T)	[45]
Progesterone Receptor (PR-A; PR-B)	GNACANNNTGTNC (PRE)	[58]
Protein 53 (p53)	CATTAG	[59]
Recombination signal Binding Protein-Jk (RBP-Jk)	(C/T)GTGGGAA	[60]
Sex-determining Region Y (SRY)	(A/T)(A/T)CAA(A/T)	[61]
Signal Transducers and Activators of Transcription (STAT)	TTCNNNNGAA	[62]
Specificity Protein (Sp-1; Sp-3)	GGGCGG (GC-box)	[22]
TATA Binding Protein (TBP)	TATAAA (TATA box)	[63]
T cell Factor 1 (TCF-1)	(G/C)ATCAAAGG	[64]
Transcription Factor II D (TFII-D)	TATAAA (TATA box)	[63]
Transcription Factor II I (TFII-I)	CANNTG	[65]
Upstream Transcription Factor 1 (USF-1)	CACGTG (E-box)	[41]
Vitamin D Receptor (VDR)	G(G/T)TCA	[66]
X-box binding protein (XBP-1)	CCACG	[67]
Yin Yang 1 (YY1)	GCCATNTT	[68]

**Table 2:** Transcription factors which have been experimentally shown to influence ERVK LTR activity

**Transcription factor**	**Cellular function**	**Implicated diseases**	**Effect on ERVK LTR**	**References**
Sp-1, Sp-3	Implicated in the regulation of genes that control multiple cellular processes, including cell cycle, apoptosis, and DNA damage.	ALS, SLE, RA Alzheimer’s Disease, Huntington’s Disease	Stimulate	[22,69-72]
YY1	Positive and negative regulator of genes involved in biological processes such as differentiation, replication, and cellular proliferation.	Cancers, SLE, neurodegeneration	Stimulate	[73-76]
NF-kB	Involved in cytoplasmic/nuclear signalling in response to stimuli such as stress, cytokines, free radicals, ultraviolet irradiation, oxidized LDL, and bacterial or viral antigens; activates transcription of a variety of genes encoding immunologically relevant proteins.	HIV infection, ALS, SLE, MS, Rheumatic disease, Cancers	Stimulate	[17,69,77-80]
NFAT-1	Plays a key role in the regulation of cytokine gene transcription during the immune response.	HIV infection, Alzheimer’s Disease, Autoimmune diseases	Stimulate	[17,81,82]
MITF-M	Induces genes essential for melanin synthesis, melanosome formation, cell cycle progression, and cell survival; essential for development of retinal pigmented epithelium and neural crest derived melanocytes.	Melanoma	Stimulate	[23]
PR	Mediates the effects of progesterone on mammary gland development.	Breast cancer	Stimulate	[83,84]
ER	Mediates the effects of estrogen on reproductive organs, bone, and brain.	SLE, Breast cancer	Stimulate	[83,85]
AR	Mediates embryonic sexual differentiation and required for maintenance of spermatogenesis	Prostate cancer, breast cancer, Kennedy’s disease	Stimulate	[86-90]

#### Influence of Sp1 and Sp3 on ERVK expression

Sp1 and Sp3 are ubiquitously expressed transcription factors, which have been pathologically implicated in cancers, neuroinflammation, and rheumatic diseases [91-93]. Induced by oxidative stress, the expression of Sp1 and Sp3 is enhanced in inflammatory diseases such as ALS and SLE [69,70,94]. Over-expression of these transcription factors may explain high levels of ERVK transcripts in these disease states, as Sp1 and Sp3 have been shown to drive ERVK expression by binding to G-rich elements, such as GC boxes found in the proviral LTR [22] (Figure 1). It has been shown that Sp1 and Sp3 mediate the transcriptional activity of the ERVK LTR as knockdown of these transcription factors using siRNA resulted in a significant loss of LTR activity [22]. Supershift assays have indicated that Sp1 and Sp3 probably bind as a heteromer to the GC boxes on the ERVK LTR, and mutation of these GC boxes resulted in downregulation of the LTR activity [22].

Several mechanisms have been depicted by which Sp1 and Sp3 may promote transcription of ERVK. Sp1 interacts with TFIID, a complex consisting of TATA binding protein (TBP) and other associated general transcription factors, and thus tethers the transcription pre-initiation complex to the TATA-independent promoter of the ERVK LTR [22]. Formation of the pre-initiation complex is a crucial step required for initiation of transcription at a promoter as it allows RNA polymerase to bind to the promoter and begin transcription. Sp1 also protects CpG islands from methylation, aiding in chromatin remodeling and creating a nucleosome free region [22], to facilitate transcription. Chromatin immunoprecipitation experiments have demonstrated that when Sp1 and Sp3 bind to adjacent nucleosomes upstream the transcription start site (Figure 1), that is, to the GC boxes 1 and 3, these regions are accessible to restriction enzymes, indicated by cleavage at these points [22]. This could only be possible if nucleosome free regions were present at the sites where Sp1 and Sp3 were bound to the LTR. Thus, expression of ERVK can be induced by the binding of Sp1 and Sp3 to the GC boxes on the TATA-independent promoter region of the ERVK LTR.

Interestingly, Sp3 may also repress ERVK expression. Since Sp3 and Sp1 are closely related and have similar affinity for the GC boxes [95], Sp3 can prevent Sp1 binding and thus may repress Sp1 mediated activation of the ERVK LTR. Sp3 has a transferable repression domain with the amino acid triplet KEE required for its repressive activity [95]. However, Sp1 also has a similar repression domain [95], yet it does not repress the activity of the LTR [22]. This implies that other characteristics of the LTR determine whether repressive action of Sp3 will occur or not. The structure and the arrangement of GC boxes on the LTR may determine whether Sp3 will repress the ERVK LTR or not. For instance, promoters with multiple binding sites often do not or weakly respond to Sp3 [95]. Since the ERVK promoter region has four GC boxes, the deactivating effect of Sp3 may be minimized. Nonetheless, the features that determine whether Sp3 acts as a repressor or activator of transcription are not well understood.

Furthermore, other members of the Sp family are also closely related to Sp3 and Sp1. These include Sp4, BTEB1, TIEG1, and TIEG2 [95]. The critical amino acids within the three zinc fingers of these members are conserved; they include KHA, RER, and RHK within the first, second, and third zinc fingers, respectively [95]. As a result, these four Sp members also recognize classical GC boxes and bind to them with a relatively similar affinity as that of Sp1 and Sp3 [95]. Thus, it can be speculated that various members of the Sp family other than Sp1 and Sp3 may also be able to induce ERVK expression by binding to the GC boxes on the LTR.

#### Influence of YY1 on ERVK expression

YY1 is a ubiquitous transcription factor, which is frequently overexpressed in cancers, degenerating neurons, and rheumatic diseases [69,73]; hence, it may be involved in causing ERVK expression documented in many inflammatory diseases. In fact, the 5′ terminus of the U3 region of the ERVK LTR binds to the YY1 enhancer complex (Figure 1). The binding of YY1 to this region, between nucleotides 62 and 83, has been shown to activate the ERVK expression in many cell lines including GH, Tera2, HepG2, and HeLa [74]. This implies that activation of ERVK LTR by YY1 may not be cell-type dependent. Mutation of this YY1 binding site has been shown to cause a 50% reduction in the activity of the ERVK LTR [74]. Moreover, addition of a functional YY1 binding site to an engineered active ERVK LTR sequence containing functional GC boxes has been observed to increase the ERVK promoter activity to 80% [22]. This indicates that in addition to essential GC boxes, transcription factor binding sites, such as those for YY1, are also crucial for activity of the ERVK LTR.

#### Influence of MITF-M on ERVK expression

Melanoma, a type of skin cancer, frequently exhibits enhanced expression of ERVK *env* and *rec* proteins [96]. Recent studies support the notion that increased ERVK expression and massive production of ERVK viral-like particles contribute to melanocyte malignancy [97]. Melanoma specific microphthalmia-associated transcription factor (MITF-M) is an oncogene of melanoma [23], and has been implicated in activating the ERVK LTR. Recently, it was shown that the ERVK-6 LTR has three MITF-M responsive sequences (E boxes) in the U3 region [23] (Figure 1), which are arranged along with TATA box and Initiator (Inr) sites in such a way that they together constitute a typical enhancer/promoter structure for RNA polymerase II found in all retroviral LTRs [23]. As a result, MITF-M is able to induce the ERVK LTR by binding to and inducing the core enhancer/promoter region.

#### Alternative transcription factors that may modulate ERVK expression

The influence of only Sp1, Sp3, YY1 and MITF-M on ERVK LTR activation has been documented. Unfortunately, the inductive or repressive ability of many other transcription factors – Oct-1, AP-1, CREB, NF-κB, IRFs, etc. (Table 1) – all of which have potentially intact binding sites on the consensus ERVK LTR (Figure 1), have yet to be studied. For example, Oct-1 and the members of the bZip family of transcription factors, AP-1 and CREB, have been shown to induce ERVK indirectly as a consequence of exogenous viral infections, which will be discussed in the following sections. Moreover, AP-1 and CREB are often over-expressed in inflammatory diseases, suggesting their potential role in disease pathogenesis by induction of ERVK. Further research is warranted in order to precisely determine the influence of these various transcription factors on the activity of ERVK LTRs.

### Putative role of interferon and inflammatory transcription factors in ERVK induction

Transcription factors associated with the innate immune response, especially NF-κB, IRF-1, IRF-3 and IRF-7, may also be able to influence the activity of the ERVK LTR. During anti-viral responses and inflammation, many of these transcription factors become up-regulated and post-translationally activated. NF-κB is known to be a key regulator of exogenous retrovirus transcription [98]. Often, oxidative stress is also implicated in the up-regulation of NF-κB in neurodegenerative and rheumatic diseases as a result of the protective cellular response [69,77,78]. Since the ERVK LTR has several NF-κB binding sites (Figure 1), this transcription factor is likely to directly influence ERVK expression.

Although inflammatory transcription factors have yet to be shown to influence ERVK LTR, they are known to control other retroviral LTRs. NF-κB binding to the HIV-1 LTR has been shown to stimulate HIV-1 production about 50-fold [99]. The members of the interferon regulatory factor (IRF) family have been shown to interact with the members of the NF-κB family; indeed, IRF-1 is required for full NF-κB transcriptional activity at the HIV-1 LTR enhancer [79]. Accordingly, overlapping binding sites for IRFs and NF-kB have been identified in HIV-1 LTR [79]. The ERVK LTR also contains overlapping binding sites for these transcription factors (Figure 1), suggesting functional commonality among LTR responsive elements in human retroviruses.

As shown in Figure 1, a conserved feature of ERVK LTRs is the presence of two ISRE-like motifs (5^′^-GAAANNGAAANN-3^′^), located at nt379 and nt563. These conserved motifs may accommodate IRF binding, in conjunction with NF-κB, fulfilling the transcriptional priming of traditional interferon-stimulated genes (reviewed in [101,102]). Of note, at both ISRE sites, the proximal GAAA half-site motifs are mutated, likely favouring IRF-7 over IRF-3 binding [103]. IFNα signalling can directly lead to IRF-7 activation [104], and several reports indicate that induction of ERVK-18 superantigen by herpesviruses in PBMC may be mediated through IFNα [105-107]. This not only establishes a link between exogenous virus infection and the induction of the ERVs, but may suggest the involvement of IRF activity in mediating ERVK transcription. To date, there is a lack of empirical evidence to support that other ERVK members are induced by anti-viral signalling pathways or activation of select IRFs.

In contrast, pro-inflammatory cytokines, such as TNFα and IL-6, have been shown to modulate ERVK transcriptional activity [11]. TNFα can engage an autocrine signalling loop that culminates in IRF-1 activation, a sustained low-level IFNβ response and IRF-7 expression in macrophages [108]. In addition, TNFα is a strong activator of NF-κB and AP-1. Together, these factors may contribute to the enhancement of ERVK expression. Similarly, IL-6 enhances IRF-1 transcription, and can affect IRF-1 DNA binding in select cell types [109]. IL-6 signalling also drives the activation of STAT3, which can bind ISRE *cis*-elements, another potential mechanism to activate the ERVK LTR.

### An extra layer of control: modulation of ERVK expression by hormonal regulation

The effect of estrogen and progesterone on ERVK expression has been exclusively studied in breast cancer tissues. Most breast cancer cell lines and many breast tumor tissues exhibit significantly higher levels of ERVK *env* expression as compared to normal breast tissues [110,111]. The expression of *env* transcripts has been shown to be up-regulated 5 to 10-fold in breast cancer cell lines upon estradiol treatment followed by progesterone [83,112], suggesting the presence of functional hormone response elements in the ERVK LTR. In fact, several estrogen, androgen and progesterone response elements (ERE, ARE and PRE, respectively) are predicted in the U3 region of the LTR (Figure 1) [83,86,112]. Besides ERVK *env* expression, enzymatically active ERVK reverse transcriptase protein has also been detected in breast tumor biopsies and the breast cancer cell line T47D [112]. Again, estradiol/progesterone treatment of T47D cells lead to an increased level of ERVK reverse transcriptase protein expression, as well as its enzymatic activity [112]. Interestingly, stimulation of ERVK expression has not been demonstrated by treatment with estradiol or progesterone alone, but specifically with estradiol followed by progesterone [83]. This suggests that estradiol has a priming effect on the ERVK LTR, whereby it may alter basal transcription factor affinity for the LTR, making it more easily accessible to progesterone-receptor complexes. Thus, these studies strongly point to the notion that steroid hormones contribute to the regulation of ERVK LTRs.

The gene expression of ERVK may not only be influenced directly by cellular and viral transcription factors, but also indirectly by various pharmaceutical agents [71,100] which act on (or counteract) the transcription factors that can bind to the LTR of ERVK. In particular, the extent of hormone-responsive elements (Figure 1; ERE, PRE, GRE) in the ERVK promoter suggests a susceptibility to the action of endocrine disruptors [113]. For example, the endocrine disrupting compound bisphenol-A (BPA) is known to mimic estrogen signalling pathway [114], and may modulate estrogen receptor targets such as ERVK. Endocrine disruption leading to ERVK re-activation may bridge the often speculative association between environmental exposures and the establishment of chronic inflammatory disease.

### Influence of viral proteins on expression of ERVK

Besides exploiting cellular transcription factors, ERVK can also utilize virally-encoded proteins for its induction. This versatility in part explains the up-regulation of ERVK by exogenous viruses such as Human Immunodeficiency Virus-1 (HIV-1), Human T-Lymphotrophic Virus-1 (HTLV-1), Herpes Simplex Virus-1 (HSV-1) and Epstein Barr Virus (EBV) [115-117]. These viruses provide viral proteins that increase the affinity of transcription factors for their binding sites on the ERVK LTR, thereby trans-activating ERVK.

#### Induction of ERVK by exogenous retroviruses

The expression of ERVK is often abnormally elevated in HIV-1 infected individuals, reflecting vastly increased viral RNA titres in their plasma [17,118,119]. However, the mechanism underlying this phenomenon had remained unknown until recently. It had long been proposed that HIV-1 proteins Vif and Tat may induce the ERVK LTR directly or indirectly [117,118,120]. HIV-1 accessory protein Vif has been shown to impair the translation of APOBEC3G mRNA and accelerate its post-translational degradation [121]. In the absence of APOBEC3G activity, there is an enhancement of *de novo* ERVK infectivity, as demonstrated experimentally *in vitro* using virions derived from reconstituted elements [29,31]. However, it remains unclear whether HIV Vif interaction with APOBEC3G exerts a regulatory effect on ERVK expression [119,122].

Moreover, the direct interaction of Tat with nascent ERVK RNA, and thus the induction of viral transcript expression, had always been suspected. It has only recently been demonstrated that HIV-1 proteins Vif and Tat independently activate ERVK expression [17,122]. Transfection of Jurkat T cells and 293FT cell lines with plasmids encoding functional Tat and Vif proteins significantly up-regulated ERVK *gag* RNA by 21- and 15-fold, respectively [17]. The levels of *rec* and *np-9* transcripts and the expression of the ERVK capsid protein also increased in the presence of Tat in several cell lines, as well as in primary lymphocytes that are major targets of HIV-1 infection [17]. Similarly, HTLV-1 Tax protein also promotes ERVK transcription in Jurkat T cells [123].

Several mechanisms have been proposed by which HIV-1 Tat may trans-activate ERVK. Tat activates transcription from the HIV-1 promoter by interacting with Cyclin T1 and recruiting the host positive transcription elongation factor b (P-TEFb) to its LTR [124]. The Tat induced activation of ERVK expression also occurs at the level of the ERVK LTR, but does not involve its interaction with Cyclin T1 or P-TEFb [17]. Mutations in the transactivation domain of Tat, which rendered it either unable to bind to Cyclin T1 or increased its binding to P-TEFb, had no measurable effect on Tat’s capacity to drive ERVK LTR activity.

HIV-1 LTR can be activated in a TAR-independent manner; this effect occurs through the interaction of Tat with Sp1 sequences in the U3 region of HIV-1 LTR as well as with NF-κB [17,98]. Thus, Tat may activate ERVK promoter by interacting with GC boxes and NF-κB. In fact, activation of the ERVK LTR by Tat has been demonstrated to be mediated by its interaction with NF-κB and NFAT-1 cellular transcription factors [17]. Chemical inhibition of NF-κB and NFAT-1 repressed Tat mediated activation of ERVK promoter, significantly diminishing Tat-mediated ERVK *gag* RNA levels. ChIP assays further confirmed that NF-κB and NFAT-1 were activated and interacted with multiple binding sites on ERVK LTR (Figure 1) in response to Tat. Interestingly, these intact and active NF-κB and NFAT-1 binding sites overlap with interferon-stimulated response elements (ISREs) in the ERVK LTR (Figure 1), suggesting the potential role of the innate immune system and associated inflammatory transcription factors in regulating the ERVK expression.

Furthermore, HIV-1 infection may also contribute to ERVK up-regulation indirectly by promoting opportunistic infections. Destruction of the immune system by HIV-1 can facilitate the replication of other viruses such as HSV-1 and HTLV-1, as well as the protozoan *Toxoplasma gondii*[125], all of which have been shown to trans-activate the ERVK LTR.

The Tax protein produced by HTLV-1 may modestly trans-activate ERVK, as demonstrated by Tax-driven activation of a luciferase reporter under the control of a TD47 ERVK LTR in Jurkat T cells [123]. The Tax protein likely increases the affinity of several transcription factors, specifically Sp1, NF-κB, c-Fos/c-Jun heterodimers (AP-1), and CREB, for their DNA binding sites on the ERVK LTR [20,98]. A caveat of these experiments is that Tax-mediated induction of ERVK has not yet been demonstrated *in vivo*, in either HTLV-1 carriers, patients with Adult T-cell Leukemia or individuals with HTLV-1 associated myelopathy (HAM/TSP).

#### Induction of ERVK by Herpesviruses

The immediate early protein, ICP0, produced by HSV-1 induces the LTR directed transcription of ERVK *in vitro*[116]. This effect is mediated through the up-regulation of AP-1 activity. Deletion analysis of various nucleotide sequences in the ERVK LTR has shown that the AP-1 binding site between the nucleotides 243 and 253 is required for ICP0 to trans-activate ERVK [116]. Destruction of this site completely abolished ICP0 responsiveness, whereas the activation of ERVK by ICP0 was not affected by deletion of any other nucleotides, such as 828–968 and the YY1 binding site [116]. HSV-1 may also induce ERVK through up-regulation of Oct-1 activity mediated by the immediate early protein 1 (IEP1) [126]. IEP1 has been shown to increase the expression of ERVW *in vitro* by facilitating the binding of Oct-1 to its binding site on the LTR [126]. In addition, IEP1 has been shown to increase the expression of ERVK [116]. Since ERVK also has Oct-1 binding sites, IEP1 of HSV-1 may induce ERVK by increasing the affinity of Oct-1 for its binding site on the ERVK LTR. However, the *in vivo* induction of ERVK by HSV-1 immediate early proteins is yet to be confirmed.

Similarly, EBV infection has been reported to stimulate the production of an ERVK-18 *env*-derived superantigen (SAg) specific to T cells that express T cell receptor β chain variable-13 (TcRβCV-13) [127], a mechanism which can enhance the pathogenicity of EBV. ERVK-18 is a classic example of an intragenic ERV, located on the anti-sense DNA strand within first intron of the cellular *cd48* gene. Trans-activation of ERVK-18 can be driven by EBV-encoded latent membrane protein LMP-2A [128,129]. A series of tyrosine mutants of LMP-2A revealed that the immunoreceptor tyrosine-based activation motif (ITAM) of LMP-2A is responsible for inducing the ERVK-18 *env* expression [129]. Furthermore, deletion of an enhancer in a 25 kb region downstream the ERVK-18 *env* gene abolished production of the SAg [129], indicating that ITAM interacts with this enhancer to induce ERVK-18 expression. A putative genomic NF-κB binding site adjacent to the provirus was identified as a potential candidate for interaction with the ITAM of LMP-2A [129]. This demonstrates not only the importance of the ERVK LTR, but also the genomic context of the ERV, in enhancing its transcription factor-mediated expression.

## Conclusion

Although several studies indicate the effects of epigenetic mechanisms in controlling ERVK expression, the literature clearly awaits more studies on tissue-specific, pro-inflammatory and hormone-regulated transcription factors that promote or repress ERVK transcription in health and inflammatory diseases. Surprisingly, very few transcription factors – Sp1, Sp3, YY1, MITF-M, and estrogen/progesterone – have been experimentally shown to induce ERVK; however, these transcription factors are not necessarily tissue-specific as they are ubiquitously expressed. Bioinformatic examination of several ERVK LTRs clearly unveils the multitude of possible binding sites for unique and ubiquitous transcription factors, but it is yet to be determined whether these sites are at all functional [24]. One clear feature of the ERVK LTRs is the two ISRE sequences, which is highly suggestive of ERVK regulation in the context of innate immune response and inflammation.

A large part of the debate surrounding a causal relationship of ERVK in disease pathology is the issue of bystander activation – advances in our understanding of the transcriptional regulation of the ERVK LTR will clarify whether ERVK expression is a consequence, cause or conjoined mechanism of inflammatory disease. Thus, we are indeed strongly in need of an ERVK Transcriptome Project [33,130,131], whereby determining the transcriptional regulation of ERVK expression and its association with inflammatory diseases will allow us to point to transcription factors as primary cellular targets for therapeutic intervention.

## Abbreviations

ALS: Amyotrophic Lateral Sclerosis; EBV: Epstein Barr Virus; ERV: Endogenous retrovirus; ERVK: Endogenous retrovirus-K; ERVW: Endogenous retrovirus-W; HIV: Human Immunodeficiency Virus; HSV-1: Herpes Simplex Virus-1; HTLV-1: Human T-Lymphotrophic Virus-1; ISRE: Interferon-stimulated response element; LTR: Long terminal repeat; NF-κB: Nuclear factor-κB; RA: Rheumatoid Arthritis; SLE: Systemic Lupus Erythematosus.

## Competing interests

The authors declare that they have no competing interests.

## Authors’ contributions

MM performed the sequence alignments and bioinformatic annotations. Both MM and RND drafted the manuscript. Both authors read and approved the final manuscript.
